# Anti-Inflammatory and Antioxidant Effects of Diosmetin-3-*O*-β-d-Glucuronide, the Main Metabolite of Diosmin: Evidence from Ex Vivo Human Skin Models

**DOI:** 10.3390/molecules28145591

**Published:** 2023-07-22

**Authors:** Sylvie Boisnic, Marie-Christine Branchet, Barbara Quioc-Salomon, Julie Doan, Catherine Delva, Célia Gendron

**Affiliations:** 1 GREDECO (Group of Research and Evaluation in Dermatology and Cosmetology), 69 Rue de la Tour, 75016 Paris, France; 2 Laboratoire Innotech International, 22 Avenue Aristide Briand, 94110 Arcueil, France; 3 Inferential, 35, Rue Godot de Mauroy, 75009 Paris, France

**Keywords:** anti-inflammatory, antioxidant, chronic venous disease, diosmin, metabolite, skin explant

## Abstract

Diosmin is used to relieve chronic venous disease (CVD) symptoms. This study aimed to investigate the anti-inflammatory and antioxidant effects of diosmetin-3-*O*-β-d-glucuronide, the major metabolite of diosmin, using human skin explants. The explants were exposed to substance P (inflammation model) or UVB irradiation (oxidative model) and to five diosmetin-3-*O*-β-d-glucuronide concentrations. Inflammation was evaluated through interleukin-8 (IL-8) secretion measurements and capillary dilation observation, and oxidation was evaluated by measuring the hydrogen peroxide levels and observing cyclobutane pyrimidine dimers (CPDs). In substance-P-exposed explants, diosmetin-3-*O*-β-d-glucuronide induced a significant decrease in IL-8 secretions, with a maximal effect at 2700 pg/mL (−49.6%), and it reduced the proportion of dilated capillaries and the mean luminal cross-sectional area (*p* < 0.0001 at all tested concentrations), indicating a vasoconstrictive effect. In UVB-irradiated fragments, diosmetin-3-*O*-β-d-glucuronide induced a significant decrease in hydrogen peroxide production and in the number of CPD-positive cells, reaching a maximal effect at the concentration of 2700 pg/mL (−48.6% and −52.0%, respectively). Diosmetin-3-*O*-β-d-glucuronide induced anti-inflammatory and antioxidant responses, with the maximal effect being reached at 2700 pg/mL and corresponding to the peak plasma concentration estimated after the oral intake of 600 mg of diosmin, the daily dose usually recommended for the treatment of CVD. These ex vivo findings suggest a protective role of diosmetin-3-*O*-β-d-glucuronide against inflammatory and oxidative stress affecting the vascular system in CVD pathophysiology.

## 1. Introduction

Chronic venous disease (CVD) is a common condition affecting up to 80% of the population worldwide [1,2,3]. It is characterized by morphological and functional abnormalities of the lower-limb venous system. CVD is manifested by symptoms such as feelings of swelling, heaviness and/or pain in the legs, and/or clinical signs such as varicose veins, leg oedema, and skin changes (e.g., pigmentation, dermatitis). At the most advanced stage, the disease may culminate in leg ulceration.

The exact aetiology of CVD remains uncertain, but structural changes in the venous wall and valves have been reported to cause venous reflux and/or obstruction, inducing venous hypertension [4]. Chronic venous hypertension and valvular incompetence induce shear stress changes in the venous wall that may lead to leucocyte recruitment and adhesion [5]. This may trigger an inflammatory cascade, potentially leading to a further increase in valvular incompetence and venous hypertension [6]. Leucocyte sequestration may also decrease local capillary perfusion and induce free radical generation, creating an oxidative environment that promotes inflammation and hinders healing [7]. Inflammation and oxidative stress have, therefore, been suggested to play a crucial role in the onset and development of CVD [8,9].

Oral venoactive drugs are used to relieve the symptoms of CVD, and diosmin, a naturally occurring flavonoid, is considered the treatment of choice [10,11]. The beneficial effects of diosmin are thought to be mediated by its anti-inflammatory properties, particularly via a reduction in leucocyte adhesion and cytokine production [9], and by its antioxidant activity, involving the scavenging of reactive oxidative species (ROS) [9,12].

**Figure 1:**
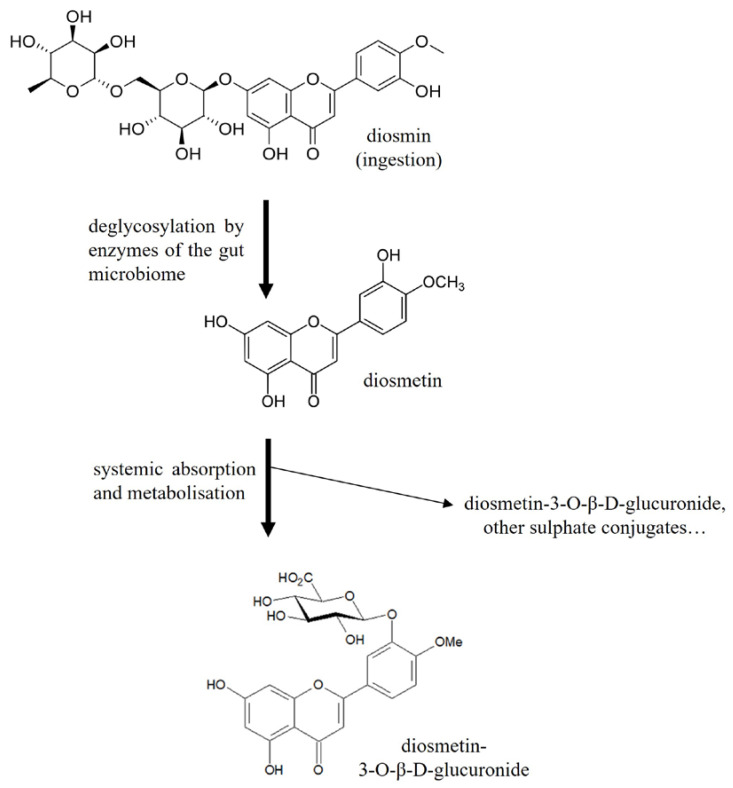
Metabolic pathway of diosmin after ingestion: diosmetin-3-*O*-β-d-glucuronide was confirmed as the major metabolite of diosmin [13].

After ingestion, diosmin is rapidly hydrolysed by enzymes of the gut microbiome to its aglycone form, free diosmetin, which is then absorbed and metabolised to glucuronide conjugates. One of these conjugates, diosmetin-3-*O*-β-d-glucuronide, has been identified as the major circulating metabolite (Figure 1) [13], and may, therefore, be involved in the pharmacological actions attributed to diosmin in CVD, although it has not been demonstrated until now.

The aim of this study was to evaluate the anti-inflammatory and antioxidant potential of diosmetin-3-*O*-β-d-glucuronide using healthy human skin samples kept alive in an organ culture as an alternative to animal models [14]. Diosmetin-3-*O*-β-d-glucuronide was added at different concentrations to the culture medium, allowing its diffusion into the dermis as in the context of the systemic administration of diosmin in humans. Two experimental models, a substance-P-induced inflammation model and an ultraviolet-B (UVB)-induced oxidative stress model, were used, as in previous experiments aiming to determine the protective effects of a 2% diosmin cream on the skin [15]. Substance P, the main neuropeptide triggering inflammatory responses in the skin, induces a composite neurogenic inflammation process involving NK1 receptors and histamine release from mast cells, leading to vasodilation, oedema, and pro-inflammatory cytokine release [16,17]. UVB irradiation induces molecular modifications, such as the creation of covalent links between two consecutive pyrimidine nucleotides on a DNA strand, forming so-called cyclobutane pyrimidine dimers (CPDs). UVB irradiation also generates ROS such as hydrogen peroxide (H_2_O_2_), creating an oxidative environment [18].

## 2. Results

### 2.1. Anti-Inflammatory Effect of Diosmetin-3-O-β-d-Glucuronide after Exposure to Substance P (SP)

**Figure 2:**
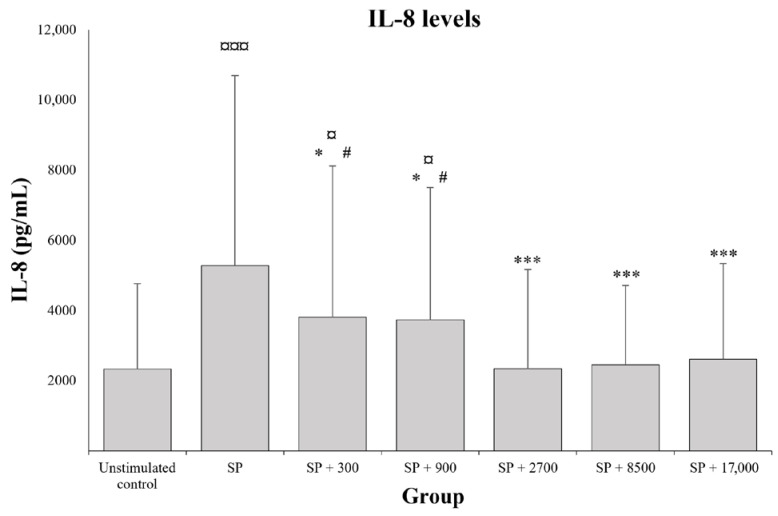
Anti-inflammatory effect of diosmetin-3-*O*-β-d-glucuronide: IL-8 levels in the supernatant of skin explant fragments (*n* = 22 fragments per group) unstimulated or stimulated by substance P (SP) and exposed to different concentrations of diosmetin-3-*O*-β-d-glucuronide (0; 300; 900; 2700; 8500; or 17,000 pg/mL). Values are expressed as the mean and bars represent the standard deviation (SD). The symbols indicate that the treatment is significantly different from the unstimulated control at ¤ *p* < 0.05 and ¤¤¤ *p* < 0.0001, significantly different from SP at * *p <* 0.05 and *** *p* < 0.0001, and significantly different from SP + 2700 at # *p* < 0.05.

Exposure to SP induced a significant increase in IL-8 secretions (*p* < 0.0001) compared with the unstimulated control (Figure 2; +192.2 ± 235.8%). The addition of diosmetin-3-*O*-β-d-glucuronide elicited a significant decrease in IL-8 levels (*p* < 0.05), ranging from −20.5% ± 35.2% to −49.6% ± 23.8%, compared with SP-stimulated skin fragments not exposed to the diosmin metabolite, and this effect reached a maximum at 2700 pg/mL of diosmetin-3-*O*-β-d-glucuronide (Figure 2). Compared with the maximal effect observed at 2700 pg/mL of diosmetin-3-*O*-β-d-glucuronide, lower diosmetin-3-*O*-β-d-glucuronide concentrations were associated with significantly higher (*p* < 0.01) IL-8 levels (SP + 300 pg/mL: +84.9 ± 128.8% and SP + 900 pg/mL: +102.6 ± 157.7% versus SP + 2700 pg/mL), with no additional effect being observed at higher concentrations (*p* > 0.05). At concentrations of 2700 pg/mL and above, the levels of secreted IL-8 were similar to that observed in the unstimulated control (*p* > 0.05).

**Figure 3:**
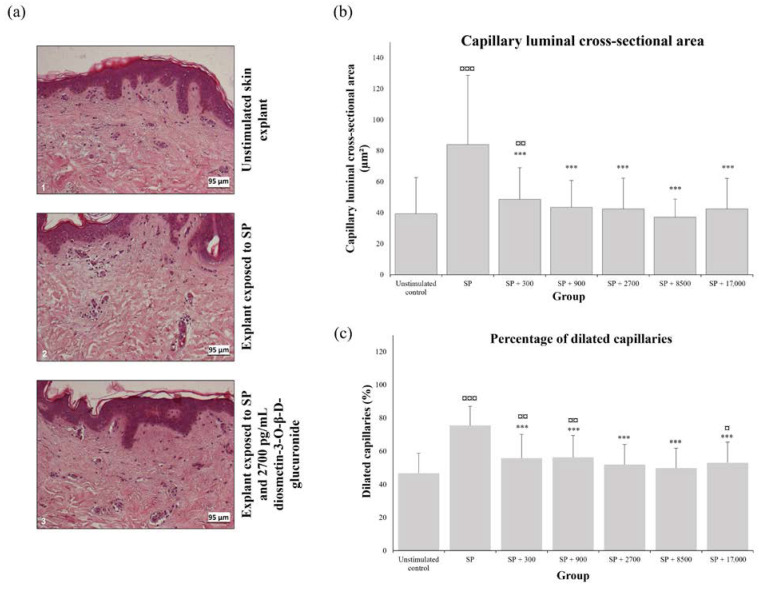
Vasoconstrictive effect of diosmetin-3-*O*-β-d-glucuronide: immunohistochemical observation of unstimulated skin explant fragments (**a1**), fragments exposed to substance P (SP) only (**a2**) or to SP and 2700 pg/mL diosmetin-3-*O*-β-d-glucuronide (**a3**), capillary luminal cross-sectional area (**b**), and percentage of dilated capillaries (**c**) in skin explants, unstimulated or stimulated by substance P (SP) and exposed to different concentrations of diosmetin-3-*O*-β-d-glucuronide. Values are expressed as the mean and bars represent the standard deviation (SD). The symbols indicate that the treatment is significantly different from the unstimulated control at ¤ *p* < 0.05, ¤¤ *p* < 0.01, and ¤¤¤ *p* < 0.0001, and significantly different from SP at *** *p* < 0.0001.

Exposure to SP resulted in vasodilation (Figure 3a), which was associated with a significant increase in the mean luminal cross-sectional area of the capillaries (Figure 3b; +141.2 ± 88.4%) and in the percentage of dilated capillaries (Figure 3c; +69.2 ± 41.3%) compared to the values determined in the unstimulated control skin fragments (*p* < 0.0001). In the presence of diosmetin-3-*O*-β-d-glucuronide, the mean luminal cross-sectional area significantly decreased from −33.8 ± 32.8% to −48.6 ± 21.4% (Figure 3b) and the percentage of dilated capillaries was reduced by −24.7 ± 22.0% to −33.5 ± 15.4% (Figure 3c) compared to that in explants treated with SP alone (*p* < 0.0001). These decreases are significantly different from SP alone starting from the first tested concentration (*p* < 0.0001). The mean luminal cross-sectional area became similar to the control at a concentration of 900 pg/mL (Figure 3b). The percentage of dilated capillaries became similar to the unstimulated control at a concentration of 2700 pg/mL of diosmetin-3-*O*-β-d-glucuronide, with the exception of the dose of 17,000 pg/mL, which resulted in a slight increase (Figure 3c).

### 2.2. Protection against Free Radical Release after UVB-Induced Skin Damage

**Figure 4:**
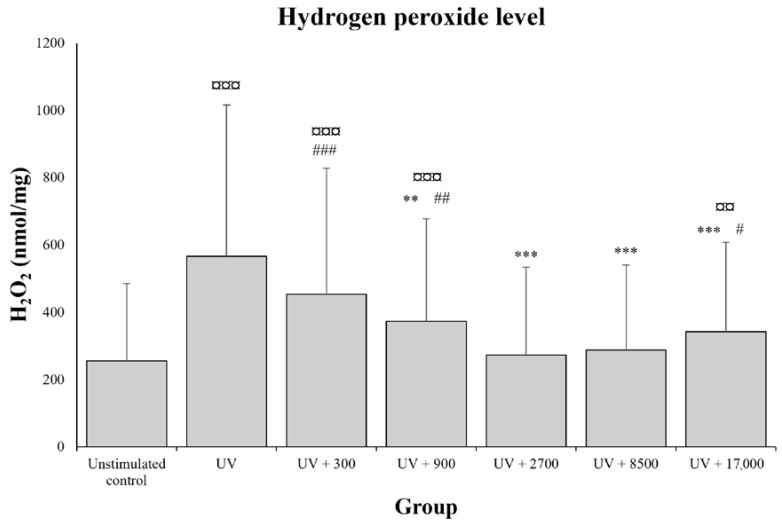
Antioxidative effect of diosmetin-3-*O*-β-d-glucuronide: quantification of hydrogen peroxide (H_2_O_2_) levels in skin explant fragments (*n* = 22 fragments per group) unstimulated (not UVB-irradiated) or exposed to UVB irradiation and different concentrations of diosmetin-3-*O*-β-d-glucuronide (0; 300; 900; 2700; 8500; or 17,000 pg/mL). Values are expressed as the mean and bars represent the standard deviation (SD). The symbols indicate that the treatment is significantly different from the unstimulated control at ¤¤ *p* < 0.005 and ¤¤¤ *p* < 0.0001, significantly different from UV at ** *p* < 0.001 and *** *p* < 0.0001, and significantly different from UV + 2700 at # *p* < 0.05, ## *p* < 0.005, and ### *p* < 0.0001.

UVB-induced oxidative stress generated a significant rise of +276.0 ± 520.7% in the hydrogen peroxide levels compared to the unstimulated control (Figure 4; *p* < 0.0001). The addition of diosmetin-3-*O*-β-d-glucuronide significantly decreased this UVB-induced release of hydrogen peroxide from a concentration of 900 pg/mL onwards (Figure 4). Compared to the skin explant fragments exposed to UVB-induced oxidative stress only, the decrease was greatest at a diosmetin-3-*O*-β-d-glucuronide concentration of 2700 pg/mL (−48.6 ± 25.5%), and no additional inhibition of hydrogen peroxide release was observed at higher concentrations (UVB + 8500 pg/mL: *p* > 0.05 versus UVB + 2700 pg/mL). The levels of hydrogen peroxide in the explant fragments exposed to 2700 and 8500 pg/mL of diosmetin-3-*O*-β-d-glucuronide did not differ from those observed in the unstimulated control (*p* > 0.05).

**Figure 5:**
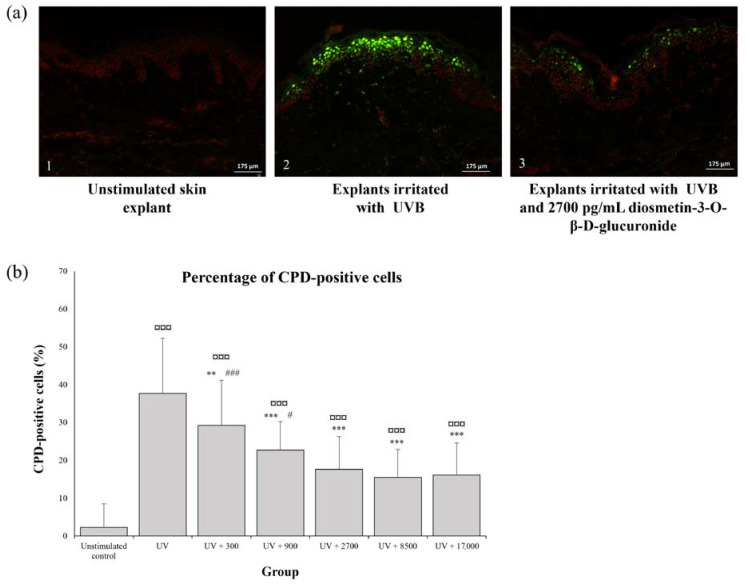
Protective effect of diosmetin-3-*O*-β-d-glucuronide against UVB-irradiation: (**a**) Immunofluorescence visualisation of cyclobutane pyrimidine dimers (CPDs) in skin explant fragments unstimulated (**a1**), exposed to UVB irradiation only (**a2**), or exposed to UVB irradiation and 2700 pg/mL of diosmetin-3-*O*-β-d-glucuronide (**a3**). (**b**) Quantification of CPD-positive cells in skin explant fragments (*n* = 22 fragments per group), unstimulated or exposed to UVB irradiation and to different concentrations of diosmetin-3-*O*-β-d-glucuronide (0; 300; 900; 2700; 8500; or 17,000 pg/mL). Values are expressed as the mean and bars represent the standard deviation (SD). The symbols indicate that the treatment is significantly different from unstimulated control at ¤¤¤ *p* < 0.0001, significantly different from UV at ** *p* < 0.0005 and *** *p* < 0.0001 and significantly different from UV + 2700 at # *p* < 0.05 and ### *p* < 0.0001.

UVB irradiation induced a marked increase in the number of CPD-positive cells, whereas CPDs were not seen in 97.7 ± 6.2% of the cells in non-irradiated explants (Figure 5a) (*p* < 0.0001). A significant decrease in the percentage of CPD-positive cells was observed in irradiated skin fragments exposed to diosmetin-3-*O*-β-d-glucuronide from the lowest tested concentration onwards (UV + 300 pg/mL: −20.7 ± 23.8% versus the positive irradiated control), with this effect reaching a plateau at concentrations ≥ 2700 pg/mL (UV + 2700 pg/mL: −52.0 ± 17.7% versus the positive control) (Figure 5b). Lower diosmetin-3-*O*-β-d-glucuronide concentrations were associated with a significantly higher (*p* < 0.01) proportion of CPD-positive cells (UVB + 300 pg/mL: +84.9 ± 81.1% and UVB + 900 pg/mL: +45.1 ± 56.9% versus UVB + 2700 pg/mL), with higher diosmetin-3-*O*-β-d-glucuronide concentrations not inducing any additional effect (*p* > 0.05) (UVB + 8500 pg/mL: −3.9 ± 39.9% and UVB + 17,000 pg/mL: −0.31 ± 38.05% versus UVB + 2700 pg/mL).

## 3. Discussion

This study is the first to focus on the protective role of diosmetin-3-*O*-β-d-glucuronide against two contributors in the pathophysiology of CVD, i.e., inflammation and oxidative stress. All previous in vitro studies have demonstrated the anti-inflammatory and antioxidative properties of diosmin or diosmetin compounds, yet none of these compounds have been detected in the plasma samples of treated patients. Notably, after ingestion, diosmin is transformed by the gut microbiome into diosmetin, which is absorbed and immediately converted to glucuronide conjugates (Figure 1). Based on the current knowledge, diosmetin-3-*O*-β-d-glucuronide appears to be the main metabolite of diosmin [13].

In this study, human skin organ culture models exposed either to substance P or UVB were used to trigger inflammatory or oxidative reactions, respectively. Using the same models, the topical application of a 2% diosmin cream was previously shown to inhibit the release of ROS in skin explant fragments exposed to UVB, to down-regulate cytokine release (IL-8), and to have vasoconstrictive effects in SP-stressed explant fragments [15]. As diosmin is mainly used as an oral treatment, it seemed important to confirm these results in a model mimicking systemic exposure to diosmin by using the major circulating metabolite of diosmin, diosmetin-3-*O*-β-d-glucuronide.

Analogous to the observations in the first study reported in [15], our results, obtained on a larger sample of skin explant fragments exposed to substance P, showed that diosmetin-3-*O*-β-d-glucuronide induces both anti-inflammatory and vasoconstrictive effects. The release of the cytokine IL-8 was concentration-dependently reduced after exposure to this metabolite of diosmetin, as previously reported with various flavonoids in lipopolysaccharide (LPS)-stimulated whole blood cell cultures, with concomitant reductions in other pro-inflammatory markers such as IL-1, TNF-α, and IL-6 [19]. It has been suggested that inflammation during CVD involves the activation of the NF-κB pathway, resulting notably in an increase in vascular endothelial growth factor (VEGF) expression in pathological venous endothelial cells [20]. The anti-inflammatory effect of diosmin and its derivative could, therefore, implicate the modulation of the NF-κB pathway and a reduction in T cell receptors, as previously proposed [21,22]. This hypothesis is supported by both experimental and clinical data. In mice exposed to lipopolysaccharides (LPSs), an oral administration of diosmin was shown to dose-dependently inhibit IκB-α and NF-κB subunit phosphorylation [21]. The inhibition of this pathway was associated with a reduction in the IL-1β, TNF-α, and NF-κB p65 pro-inflammatory cytokines as well as in the number of CD4+ and CD8+ T lymphocytes. Likewise, in patients suffering from chronic venous insufficiency and treated for three months with diosmin at a total daily dose of 1200 mg, a decrease in the proinflammatory factors TNF-α, IL-6, VEGF-A, and VEGF-C, which were associated with a reduction in mean leg circumference, was observed [23].

The persistent inflammatory environment associated with CVD extends to the microvascular system, in which capillaries may become markedly dilated, elongated, and tortuous. Flavonoids may reduce the related symptoms through their venoactive action [8,24]. The venotonic activity of diosmin was first demonstrated ex vivo in veins isolated from rats, which exhibited an increased amplitude of Ca^2+^-induced contractions when exposed to this compound [25]. This effect was recently confirmed in another ex vivo study, in which an increase in the Ca^2+^-dependent contractions of rat mesenteric vessels was observed after incubation with a mixture of three flavonoids, namely micronized diosmin, troxerutin, and horse chestnut extract [26]. In our study, we demonstrated in a normal skin tissue model, rather than on an isolated vein, a vasoconstrictive effect of diosmin’s main metabolite on the microvessels affected in the early stages of CVD [27]. Although it is currently unclear whether the venous symptoms are related to damage to the microcirculation, a link between the patterns of capillary impairment and the clinical class of CVD according to the CEAP (clinical–etiology–anatomy–pathophysiology) classification has been demonstrated, suggesting a role of microcirculation in symptom occurrence [27].

The inflammatory burden of CVD is also accompanied by a prooxidative environment, which, in turn, leads to further inflammation, creating a loop of continuous oxidation, inflammation, and endothelial damage [6]. Flavonoids are known to mediate ROS scavenging mechanisms and to inhibit the generation of ROS [28,29]. We similarly observed an antioxidative activity of diosmetin-3-*O*-β-d-glucuronide, expressed by a reduction in hydrogen peroxide levels and the inhibition of CPD formation. The antioxidant effect of diosmetin was previously demonstrated in an erythrocyte haemolysis assay, whereby the incubation of erythrocytes with diosmetin led to a decrease in the ROS concentration [30]. The antioxidative properties of diosmin and its metabolites may be mediated by the modulation of enzymes involved in ROS production rather than through direct ROS scavenging, as seen with other flavonoids, as a recent study showed only a moderate in vitro activity of diosmin and diosmetin on free-radical scavenging [31]. Notably, in a rodent model of alloxan-induced diabetic nephropathy, diosmin at 50 and 100 mg/kg administered per os was shown to significantly increase the activity of superoxide dismutase, catalase, and glutathione [22]. These results were recently confirmed in vitro in a hydrogen-peroxide-induced oxidative stress model in endothelial cells, where an increase in these enzymes’ activities was correlated with a decrease in the malondialdehyde concentration, a marker of oxidative stress, after exposition to diosmin and diosmetin [32]. This cytoprotective effect could also be mediated by the modulation of mitochondrial ROS production, a major source of ROS, as postulated for other flavonoids [29]. The efficacy of an oral diosmin treatment in reducing oxidative stress was also shown clinically in CVD patients receiving diosmin at 600 mg twice a day for three months, in whom the plasma levels of isoprostanes, generated by free-radical-catalysed reactions, were significantly reduced after treatment [12].

Both the anti-inflammatory and antioxidant effects of the major diosmin metabolite diosmetin-3-*O*-β-d-glucuronide could be related to its chemical structure, as many studies have shown that the structure of a given flavonoid is a crucial determinant of its function and properties. In particular, the antioxidant properties of flavonoids have been postulated to depend on the number of hydroxyl groups in their structure [33]. After conjugation, diosmetin-3-*O*-β-d-glucuronide acquires three additional hydroxyl groups (Figure 1), and this might enhance its antioxidant properties compared to non-conjugated metabolites. Further investigations comparing the properties of this specific conjugate with those of non-conjugated metabolites of diosmetin would be needed to confirm this hypothesis.

The main limitation of our study is its ex vivo nature, implying the lack of a blood supply to the skin fragments tested and the absence of surrounding tissues. Nevertheless, the results obtained are consistent with those obtained in various studies conducted in vitro [25,26,30,33], in animal models [21,22,26], and in patients [12,23], showing positive effects of diosmin or diosmetin on inflammation, oxidative stress, and vein wall contractibility. Furthermore, consistency between the ex vivo results obtained in our models and clinical findings was observed in a previous study, in which a 0.1% retinaldehyde gel reduced keratinization in 80% of the mucosal explant fragments originating from patients suffering from lichen planus, and later in 82% of patients clinically treated with the same gel [34]. Another limitation of our study is that it focused solely on the skin microvascular system, making it difficult to anticipate the likely protective effects of diosmetin-3-*O*-β-d-glucuronide in larger veins. To attempt to overcome these limitations and corroborate these ex vivo results, an in vitro “vein-on-chip” model based on “organ-on-chip” technology, which mimics the physiological conditions and recreates the vein environment and the haemodynamic forces of blood circulation, could be used as another alternative to animal experimentation [35,36,37].

Despite these limitations, it is worth noting that maximal anti-inflammatory and antioxidant effects were observed at a diosmetin-3-*O*-β-d-glucuronide concentration corresponding to the estimated level reached after a single intake of 600 mg of diosmin (2700 pg/mL). This finding is consistent with the results of a recent non-inferiority clinical trial showing that treatment with 600 mg of diosmin (Flebodia^®^) per day for six months was not inferior, with respect to alleviating CVD symptoms, to a treatment with 1000 mg of a micronized purified flavonoid fraction (Daflon^®^), corresponding approximately to the 17,000 pg/mL concentration of diosmetin-3-*O*-β-d-glucuronide tested [38]. Previous clinical studies similarly concluded the equivalent efficacy of these two diosmin-containing phlebotropic drugs [10,39,40]. All these findings support the hypothesis that increasing the diosmin dosage or using micronized diosmin may not provide further relief of CVD symptoms.

## 4. Materials and Methods

### 4.1. Culture of Human Skin Explants

Healthy human skin explants were obtained from 22 adult donors undergoing plastic surgery (abdominoplasty, *n* = 19; mammoplasty, *n* = 3; mean age: 44.6 ± 12.3 years). Informed consent was obtained from each donor prior to the study initiation.

**Figure 6:**
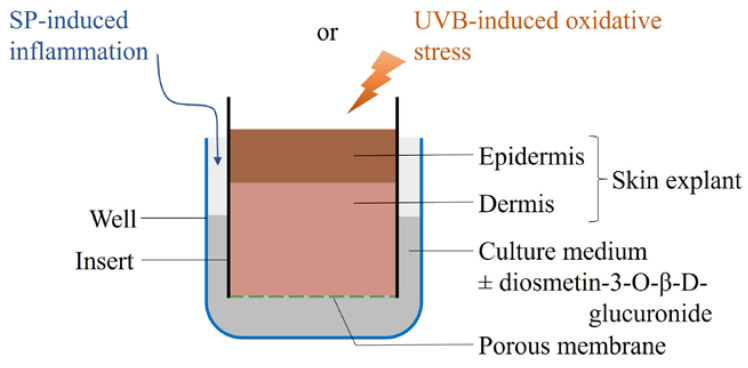
Experimental model: skin explant fragments were placed with the epidermis facing upward on the porous membrane of tissue culture inserts set in wells containing culture medium. The explant fragments were exposed to either substance P (SP) or UVB, and diosmetin-3-*O*-β-d-glucuronide was then added to the culture medium in different concentrations.

Within one hour after excision, the skin explants were rinsed with an antibiotic-containing phosphate buffer solution and divided into fragments allocated to the different experimental conditions. The fragments were placed with the epidermis facing upward on a 3 µm-pore polycarbonate membrane of tissue culture inserts set in wells of 12 mm in diameter in 12-well culture plates (Costar, VWR, Fontenay-sous-Bois, France). The culture medium (Dulbecco’s modified minimum essential medium, GlutaMax^TM^, Gibco BRL, Waltham, MA, USA) was supplemented with 0.5% foetal calf serum (DAP), 25 µg/mL of bovine pituitary extract (Gibco BRL), 50 µg/mL of hydrocortisone (H4001, Sigma-Aldrich, Saint Quentin Fallavier, France), 100 µg/mL of penicillin, and 100 µg/mL of streptomycin (Gibco BRL) and added at the bottom of the wells, allowing diffusion between the two compartments separated by the porous membrane (3 µm) (Figure 6). The culture plates were maintained at 37 °C in a humidified incubator and a 5% CO_2_ atmosphere.

The skin fragments were cultured for three days, corresponding to the time during which they were in contact with diosmetin-3-*O*-β-d-glucuronide, according to the experimental protocol described in the sections “Substance-P-induced inflammation model” and “UVB-induced oxidative stress model” below.

### 4.2. Diosmetin-3-O-β-d-Glucuronide

Diosmetin-3-*O*-β-d-glucuronide (2 mg/mL in dimethylsulphoxide, Syncom, Groningen, The Netherlands) was added to the culture medium (Figure 6) at one of five different concentrations (300; 900; 2700; 8500; or 17,000 pg/mL) once daily on D0, D1, and D2, at the time of culture medium renewal. This range of concentrations encompassed the likely diosmetin-3-*O*-β-d-glucuronide concentrations present in human plasma after the oral intake of 600 mg of diosmin (Diovenor^®^, Phlebodia^®^, or Flebodia^®^, Innothera, Arcueil, France) (2700 pg/mL), 450 mg of micronized diosmin (8500 pg/mL), or 900 mg of micronized diosmin (17,000 pg/mL), based on previous unpublished and published studies [11].

### 4.3. Substance-P-Induced Inflammation Model

#### 4.3.1. Neurogenic Inflammation Induction by Substance P

Substance P (SP), the main neuropeptide triggering inflammatory responses in the skin, was used to reproduce the neurogenic inflammation process in the cultured human skin explant fragments. After the re-equilibration of these fragments for one hour, the culture medium was renewed and supplemented with 10 µM SP (ref. H-1890, Bachem, Bubendorf, Switzerland), and diosmetin-3-*O*-β-d-glucuronide was immediately added. On day 1 (D1), the culture medium was renewed with the addition of 10 µM substance P and diosmetin-3-*O*-β-d-glucuronide. On D2, the culture medium was renewed with diosmetin-3-*O*-β-d-glucuronide-containing medium. The cultures were stopped on D3.

Each explant was divided into several fragments of equal weight for the culture, with two constituting an unstimulated control (−SP/−diosmetin-3-*O*-β-d-glucuronide) and a stress-factor-only control (+SP/−diosmetin-3-*O*-β-d-glucuronide) and the others being exposed to SP and one of the five tested concentrations of diosmetin-3-*O*-β-d-glucuronide (+SP/+diosmetin-3-*O*-β-d-glucuronide).

#### 4.3.2. Interleukin-8 Quantification

The culture medium supernatants were kept at −32 °C for interleukin-8 (IL-8) quantification. The levels of this pro-inflammatory mediator were measured using a human IL-8 ELISA kit (Human IL-8/CXCL8 DuoSet ELISA, Bio-Techne, Minneapolis, MN, USA) according to the manufacturer’s specifications. The results are expressed in pg/mL.

#### 4.3.3. Capillary Dilation Analysis

The skin explant fragments were fixed in formol for 24 h and embedded in paraffin. Serial vertical sections of 4 µm in thickness were prepared using a standard microtome and placed on albumin-coated glass slides. After haematoxylin–eosin staining, images were captured using a microscope (Olympus^®^ BX41, Olympus France, Rungis, France) with a ×40 objective coupled with a camera (QImaging Retiga SP 2000R, QImaging, Surrey, BC, Canada). The percentage of dilated capillaries was estimated in 16 fields of vision. The luminal cross-sectional area (in µm^2^) of the capillaries in the dermis was determined using an image analyser (Image Pro Plus software, v6.3.0.5012, Media Cybernetics Inc., Rockville, MD, USA).

### 4.4. UVB-Induced Oxidative Stress Model

#### 4.4.1. Oxidative Damage Induction by UVB Irradiation

The skin explant fragments were irradiated on D0 with a UVB source (Vilber Lourmat simulator, emission peak: 312 nm, composed of Vilber Lourmat T-20.L-312 mercury vapor tubes, low pressure and hot cathodes with a Vilber Lourmat RMX-365/312 radiometer; Vilber Lourmat, Collégien, France) at a dose of 6 J/cm^2^. Immediately after irradiation, diosmetin-3-*O*-β-d-glucuronide was added. The culture medium was renewed on D1 and D2 and diosmetin-3-*O*-β-d-glucuronide was added. The cultures were stopped on D3 and each UVB-exposed skin explant fragment was divided in half. One half was frozen for hydrogen peroxide quantification and the other half was fixed in formol and frozen in embedding media (OCT compound) for CPD detection.

Each explant was divided into fragments of equal weight, with two fragments constituting an unstimulated control (-UVB/-diosmetin-3-*O*-β-d-glucuronide) and a stress-factor-only control (+UVB/-diosmetin-3-*O*-β-d-glucuronide) and the other fragments being exposed to UVB and one of the five concentrations of diosmetin-3-*O*-β-d-glucuronide tested (+UVB/+diosmetin-3-*O*-β-d-glucuronide).

#### 4.4.2. Immunofluorescent Detection of Cyclobutane Pyrimidine Dimers (CPDs)

Serial 8 µm vertical sections of the frozen fragments were prepared using a microtome and placed on albumin-coated glass slides. Cells were incubated for one hour with an anti-thymine dimer antibody (mouse monoclonal, H3 Clone, ref. ab10347; Abcam, Cambridge, UK). A fluorescent secondary antibody was applied for 30 min (Alexa Fluor 488, goat anti-mouse IgG1, ref 1071-30, Clinisciences, Nanterre, France). Nuclei were stained using propidium iodide. Images were captured using a microscope (Olympus^®^ BX41Olympus) with a ×40 objective coupled with a camera (QImaging Retiga SP 2000R, QImaging). The percentage of CPD-positive cells was calculated in 10 microscope fields based on the total number of epidermal cells.

#### 4.4.3. Hydrogen Peroxide Assay

Skin fragments were lysed using a lysis buffer (0.5 M tris-HCl, pH of 7.56, 0.1% Triton X100). Hydrogen peroxide (H_2_O_2_) concentrations were measured in the lysates using the ferrous oxidation–xylenol orange (FOX) reagent (Sigma-Aldrich). This assay method is based on the oxidation of ferrous ions (Fe^2+^) to ferric ions (Fe^3+^) by hydrogen peroxide and the reaction of these ions with xylenol orange to produce a blue-purple complex measurable by spectrophotometry at 560 nm. The amount of hydrogen peroxide present in the sample was determined by comparison with a standard curve generated with cumene hydroperoxide (1–150 mM). The results are expressed in µmoles of hydrogen peroxide per mg of tissue.

### 4.5. Statistical Analysis

The results obtained for each parameter are expressed as the mean ± standard deviation (SD) of the individual values determined for the 22 skin fragments in each treatment group.

For each parameter, the percentage variations in the groups exposed to diosmetin-3-*O*-β-d-glucuronide versus the stress-factor-only control group (+substance P or +UVB/-diosmetin-3-*O*-β-d-glucuronide) were calculated for each donor. Comparisons between groups were performed using ANOVA, with the group as a fixed effect and the skin fragment as a random effect (the normality of the residual of the analysis of variance was verified by a Shapiro–Wilk test at the 1% threshold). Contrasts versus the stress-factor-only control group were evaluated. The threshold of statistical significance was set at 5%. Comparisons of each diosmetin-3-*O*-β-d-glucuronide concentration versus the unstimulated control (-SP or -UVB/-diosmetin-3-*O*-β-d-glucuronide) and the diosmetin-3-*O*-β-d-glucuronide concentration of 8500 pg/mL versus 17,000 pg/mL were made using the same methodology.

In view of the exploratory nature of the study, no adjustments for multiple comparisons were made.

Analyses were performed using SAS software (version 9.4, SAS Institute Inc., Cary, NC, USA).

## 5. Conclusions

In conclusion, our ex vivo data demonstrate for the first time that diosmetin-3-*O*-β-d-glucuronide, recognized as the major metabolite of diosmin, displays anti-inflammatory and antioxidant effects, starting from the concentration expected to be reached after the oral administration of the recommended daily dose of diosmin to manage CVD (600 mg). These effects observed in human skin explant fragments may further contribute to the understanding of the mechanisms of action of diosmin underlying CVD symptom relief.

## Data Availability

The data presented in this study are available on request from the corresponding author.

## Sample Availability

Not available.
